# Genotype-Specific Responses to Drought During Seed Production in Carrot: Biochemical, Physiological, and Seed Quality Evaluation

**DOI:** 10.3390/ijms262110642

**Published:** 2025-10-31

**Authors:** Barbara Jagosz, Małgorzata Czernicka, Iwona Kamińska, Emilia Wilmowicz, Agata Kućko, Sylwester Smoleń, Małgorzata Kapusta, Joanna Kocięcka, Stanisław Rolbiecki, Roman Rolbiecki, Leszek Róg

**Affiliations:** 1 Department of Plant Biology and Biotechnology, University of Agriculture in Krakow, al. Mickiewicza 21, 31-120 Krakow, Poland; sylwester.smolen@urk.edu.pl; 2 Department of Botany, Physiology and Plant Protection, University of Agriculture in Krakow, al. Mickiewicza 21, 31-120 Krakow, Poland; iwona.kaminska@urk.edu.pl; 3 Department of Plant Physiology and Biotechnology, Nicolaus Copernicus University, Lwowska 1, 87-100 Toruń, Poland; emwil@umk.pl; 4 Department of Plant Physiology, Institute of Biology, Warsaw University of Life Sciences-SGGW, Nowoursynowska 159, 02-776 Warsaw, Poland; agata_kucko@sggw.edu.pl; 5 Bioimaging Laboratory, University of Gdańsk, Wita Stwosza 59, 80-308 Gdańsk, Poland; malgorzata.kapusta@ug.edu.pl; 6 Department of Land Improvement, Environmental Development and Spatial Management, Poznan University of Life Sciences, Wojska Polskiego 28, 60-637 Poznań, Poland; joanna.kociecka@up.poznan.pl; 7 Department of Biogeochemistry, Soil Science, Irrigation and Drainage, Bydgoszcz University of Science and Technology, Bernardynska 6, 85-029 Bydgoszcz, Poland; rolbs@pbs.edu.pl (S.R.); rolbr@pbs.edu.pl (R.R.); 8 PlantiCo Zielonki Sp. z o.o., Zielonki Parcela, Parkowa 1A, 05-082 Stare Babice, Poland; lrog@plantico.pl

**Keywords:** carotenoids, catalase, chlorophylls, *Daucus carota* L., glutathione reductase, guaiacol peroxidase, hydrogen peroxide, MDA, proline

## Abstract

Drought stress during the reproductive phase substantially reduces seed yield and quality, posing a major challenge to sustainable crop production under climate change. This study investigated the effects of drought stress at the flowering stage on selected biochemical and physiological parameters in 18 carrot accessions. To describe the long-term consequences of drought comprehensively, we examined seed quality parameters. Our analyses revealed that stress responses are highly dependent on the genotype and the parameter examined. Regarding antioxidant responses and potential tissue damage caused by drought, ‘Dolanka’, DC97, DC265, DC359, DC522, DC701, DC704, and DC720 exhibited the highest tolerance. The photosynthetic apparatus and pigments were maintained under stress in DC233, DC522, DC717, and DC728. Germination parameters served as reliable indicators of stress tolerance in DC97, DC359, DC432, DC522, DC701, and DC722 accessions. Based on these findings and detailed discussion of the results, we conclude that tolerance/sensitivity assessment of carrot genotypes should consider the holistic response of the plant rather than individual parameters. Through overall assessment, we recommended DC522 accession as the most drought-tolerant, given its enhanced ROS (Reactive Oxygen Species) scavenging mechanisms, increased chloroplast pigments accumulation, and superior germination parameters under drought conditions. Conversely, DC295 should not be cultivated under water-deficient conditions due to its impaired ability to detoxify ROS, altered photosynthetic activity, and disrupted seed germination under such conditions. These results collectively highlight the potential for selecting drought-tolerant carrot genotypes in breeding programs targeting improved seed performance under water-limited conditions, thereby supporting the development of resilient cultivars adapted to future climate challenges.

## 1. Introduction

Carrot (*Daucus carota* L.) is one of the most widely cultivated vegetable crops worldwide due to its high nutritional value. Rich in beta-carotene, fiber, vitamins, and minerals, carrots are consumed both fresh and processed, forming an important component of the human diet [1,2,3,4,5]. Globally, carrot cultivation spans approximately one million hectares, with major producers including China (accounting for nearly 50% of global output), followed by Uzbekistan, Russia, and the United States. However, carrot production is increasingly threatened by drought stress, a key manifestation of ongoing climate change. In Poland, for example, carrot yields have declined significantly in recent years—from approximately 827,000 tons in 2017 to around 580,000 tons in 2023 [6,7,8,9]. According to the Intergovernmental Panel on Climate Change (IPCC) Sixth Assessment Report, the global mean surface temperature is projected to increase by up to 4 °C by the end of the 21st century (2081–2100) under high-emission scenarios, accompanied by increasingly irregular precipitation patterns, including intense rainfall followed by prolonged droughts [10,11,12,13,14,15,16]. These shifts are already placing stress on agricultural water supplies, increasing the need for irrigation and more efficient water management strategies [17,18,19,20]. Consequently, these climatic trends necessitate the development of improved cultivation strategies for drought-resilient crops such as carrot.

Carrot seed production is particularly vulnerable to drought, as reproductive stages—including flowering, pollination, and seed development—are highly sensitive to temperature and water availability [21,22,23]. During reproductive developmental stages, drought is particularly detrimental because it disrupts critical processes, including flowering, pollination, and seed set—all of which depend on consistent water availability. Reduced translocation of assimilates during drought conditions results in decreased seed number, weight, and compromises their quality [24,25,26,27,28,29,30,31,32]. Moreover, water deficits impair seed filling and post-set water accumulation, negatively affecting germination capacity (GC), germination index (GI), and hydrolytic enzyme activation [32]. This can lead to prolonged mean germination time (MGT), decrease the coefficient of uniformity of germination (CUG), potentially leading to abnormal seedling (AS) development reflecting less synchronized and more spread-out germination events [33,34].

Plants respond to drought stress through a range of physiological, biochemical, and morphological mechanisms, including reduced biomass accumulation, stunted growth, and consequently decreased yields [24,25,26,27,29,35,36,37,38,39,40]. Drought typically reduces leaf area, stem diameter, and leaf number, while enhancing root growth to improve water uptake [27,41]. Stomatal closure is an immediate drought response aimed at reducing transpiration; however, this also restricts CO_2_ intake, ultimately decreasing photosynthesis [25,42]. At the cellular level, drought stress triggers the accumulation of reactive oxygen species (ROS), particularly H_2_O_2_, leading to oxidative damage [43]. To counter this, plants activate antioxidant enzymes such as peroxidase (POX), catalase (CAT), and superoxide dismutase (SOD) to protect cell membranes from lipid peroxidation [44,45]. Concurrently, osmoprotectants like proline and glycine betaine are accumulated to maintain cellular turgor and membrane stability [37,46,47,48,49].

While previous studies in carrot have primarily focused on drought effects during the vegetative phase—including morphological, physiological, biochemical, and molecular changes, hormonal regulation, enzymatic activity, and the effects of water deficit on growth, yield, and root quality [50,51,52,53,54]—knowledge regarding the impact of water deficit on the reproductive stage remains scarce. This developmental phase is often the most drought-sensitive, as stress occurring during flowering and seed development can strongly affect pollination efficiency, assimilate partitioning, and final seed quality. Therefore, investigating physiological and biochemical mechanisms underlying drought responses during the reproductive phase is crucial for improving seed yield and quality and for identifying drought-tolerant breeding lines.

A deeper understanding of carrot responses to drought during seed development is essential for breeding drought-resilient cultivars. While numerous studies have explored drought effects during the vegetative phase, little is known about its impact on carrot seed quality and physiology [55]. This knowledge gap limits the identification of genotypes that can maintain reproductive success under water-limited conditions. Investigating genotypic variation can facilitate the identification of carrot breeding lines that maintain superior productivity and seed quality under limited water availability. Thus, this study aimed to evaluate the biochemical, physiological, photosynthetic, and germination responses of 18 carrot genotypes under drought conditions during the seed production phase in a controlled greenhouse environment. We hypothesized that genotypes would exhibit varying degrees of drought tolerance, which could inform breeding programs focused on resilient seed production systems.

## 2. Results

### 2.1. Biochemical Response of Carrot Plants to Drought

**Figure 1:**
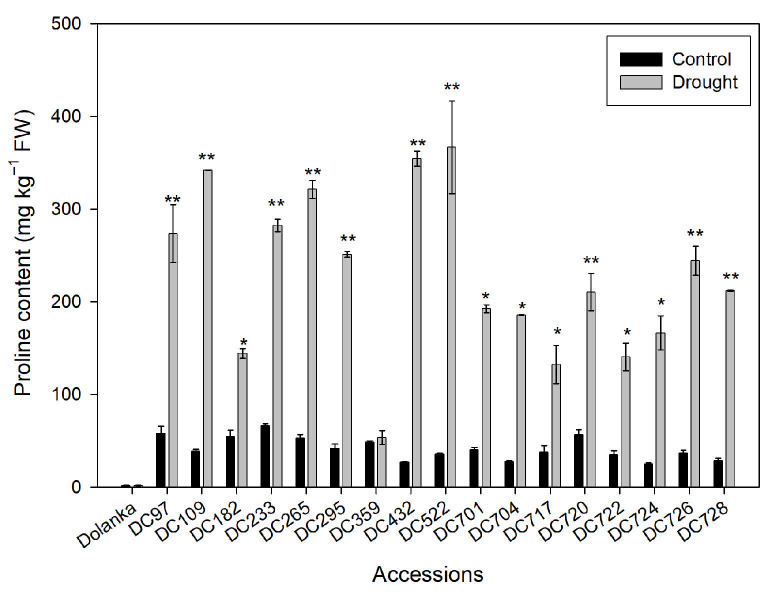
Proline content in the leaves of 18 analyzed carrot accessions grown under control and drought conditions. The amino acid concentration was measured in leaves of 150-day-old plants. Asterisks indicate significant differences between control and drought treatments (* *p* ≤ 0.05; ** *p* ≤ 0.01).

Proline accumulation was assessed in 18 carrot genotypes under well-watered (control) and drought conditions (Figure 1). Drought stress triggered a pronounced, genotype-specific increase in proline content. Under drought conditions, proline concentrations ranged from approximately 140 mg kg^−1^ FW in DC182 to ~360–400 mg kg^−^^1^ FW in DC432 and DC522. The largest absolute increases were observed in DC522 (from ~35 mg kg^−1^ to ~370 mg kg^−1^) and DC432 (~30 mg kg^−1^ to ~350 mg kg^−1^ FW). Moderate increases (150–250 mg kg^−1^ FW) were noted in DC182, DC701, DC704, DC717, DC722, and DC724 accessions. Several genotypes, including DC720, DC726, and DC728, also showed statistically significant increases in proline levels (** *p* ≤ 0.01) under drought stress. In contrast, ‘Dolanka’ and DC359 exhibited no significant change in proline content compared to the control, suggesting limited activation of osmoprotective mechanisms and poor osmotic adjustment.

**Figure 2:**
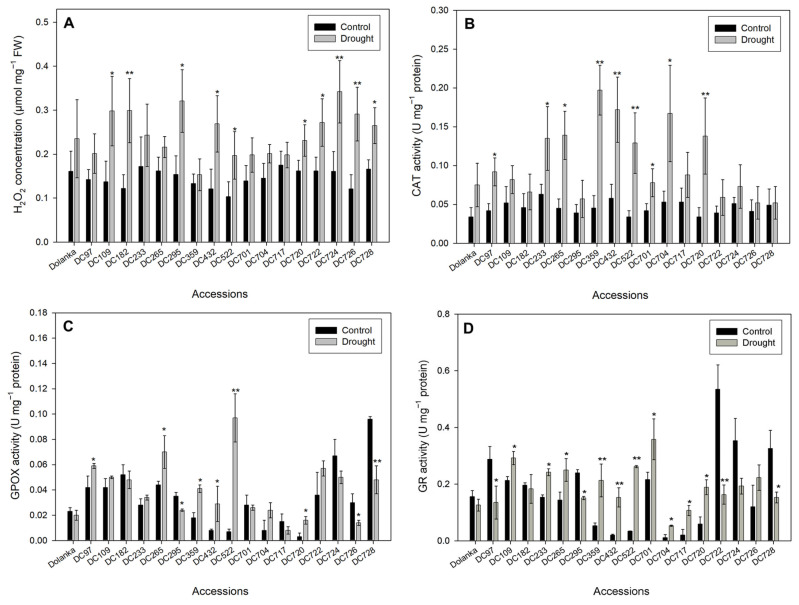
Redox-related events in the leaves of 18 analyzed carrot genotypes grown under control and drought conditions. (**A**) The content of hydrogen peroxide (H_2_O_2_), the activity of (**B**) catalase (CAT), (**C**) glutathione reductase (GR), (**D**) guaiacol peroxidase (GPOX), and were analyzed in the 150-day-old plants. Statistically significant differences in stressed plants compared to controls are ** *p* ≤ 0.01 and * *p* ≤ 0.05.

To further examine the impact of drought on various genotypes of carrot, we analyzed leaf hydrogen peroxide (H_2_O_2_) levels and the activity of reactive oxygen species (ROS) scavenging enzymes. The genotypes exhibited varying sensitivities to drought-induced oxidative stress, as reflected in changes in H_2_O_2_ content (Figure 2A) and antioxidant enzyme activities, including catalase (CAT), guaiacol peroxidase (GPOX), and glutathione reductase (GR) (Figure 2B–D), which protect plant tissues from ROS toxicity. Under drought conditions, the largest increases in leaf H_2_O_2_ levels relative to controls were observed in DC182, DC724, and DC726 genotypes, indicating high sensitivity to water deficit. Moderate but significant elevations were also detected in DC109, DC295, DC432, DC522, DC720, DC722, and DC728 (Figure 2A). In contrast, no significant changes were observed in ‘Dolanka’, DC97, DC233, DC265, DC359, DC701, DC704, and DC717, suggesting these accessions maintain oxidative balance under drought conditions. However, H_2_O_2_ accumulation did not always correlate with CAT activity (Figure 2B). The strongest CAT induction was observed in DC359, DC432, DC522, and DC720. Moderate but significant increases were also noted in DC97, DC233, DC265, DC701, and DC704. CAT activity remained unchanged in ‘Dolanka’, DC109, DC182, DC295, DC717, DC722, DC724, DC726, and DC728, potentially indicating limited enzymatic defense against H_2_O_2_ toxicity in these genotypes. Notably, DC522 exhibited more than a 100-fold increase in GPOX activity (Figure 2C), which may be a hallmark of drought tolerance. Moderate increases in GPOX activity were also recorded in DC97, DC265, DC359, DC432, and DC720. Conversely, a significant decrease in GPOX activity was found in DC295, DC726, and DC728, indicating a weakened antioxidant response. GR activity increased in DC109, DC233, DC265, DC359, DC432, DC522, DC701, DC704, DC717, and DC720, with the largest increases observed in DC359, DC432, and DC522 (Figure 2D). This suggests these genotypes maintain sufficient glutathione (GSH) pools for efficient ROS detoxification. In contrast, GR activity was inhibited in DC97, DC295, DC722, DC724, and DC728, with the most pronounced reduction seen in DC722, indicating a possible disruption of the GSH-based antioxidant defense system. No significant changes in GR activity were observed in ‘Dolanka’, DC182, and DC726.

**Figure 3:**
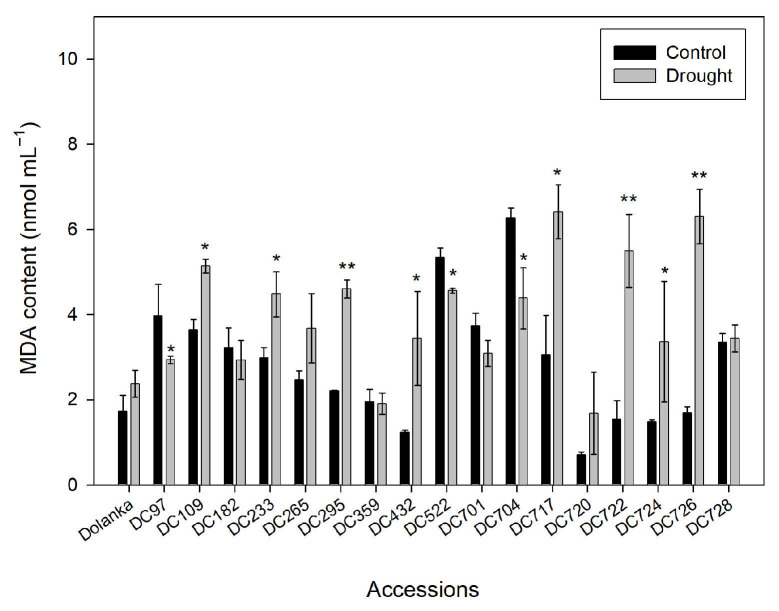
Malondialdehyde (MDA) content in the leaves of 18 carrot genotypes grown under control and drought conditions. Analyses were performed on 150-day-old plants. Asterisks indicate statistically significant differences between drought-treated and control plants (* *p* ≤ 0.05; ** *p* ≤ 0.01).

The subsequent analysis focused on malondialdehyde (MDA) content, a widely recognized marker of lipid peroxidation and oxidative stress (Figure 3). Most accessions exhibited increased leaf MDA levels under drought, indicating enhanced oxidative damage to cellular membranes. Statistically significant increases were observed in DC109, DC233, DC265, DC295, DC359, DC432, DC522, DC717, DC722, DC724, and DC726, with the highest MDA accumulation (~6 nmol mL^−1^) recorded in DC717 and DC726. In contrast, ‘Dolanka’, DC182, DC701, DC720, and DC728 maintained stable MDA levels, suggesting superior membrane stability and oxidative stress tolerance. Remarkably, DC97 showed a significant decrease in MDA content under drought conditions, which may reflect enhanced antioxidative protection and membrane integrity.

### 2.2. Photosynthetic Apparatus Response of Carrot to Drought

**Figure 4:**
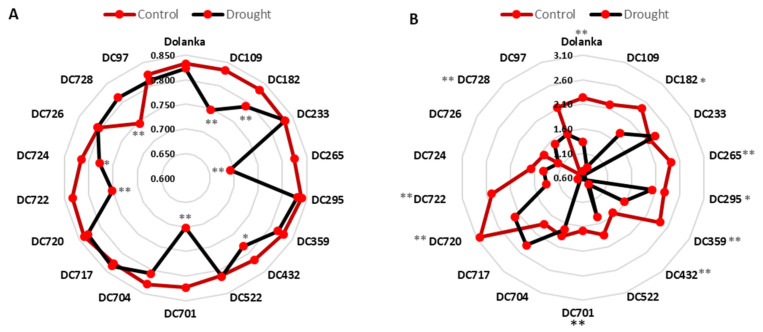
Maximum quantum yield of photosystem II (**A**, Fv/Fm) and (**B**) Performance Index (PI) in the leaves of 18 carrot genotypes cultivated under control and drought stress conditions. Measurements were performed on 150-day-old plants. Statistically significant differences in stressed plants compared to controls are * *p* ≤ 0.1 and ** *p* ≤ 0.05.

Considering the condition of the photosynthetic apparatus, the tested carrot genotypes differentially responded to drought. Under control conditions, the maximum quantum yield of photosystem II (Fv/Fm) varied among genotypes, ranging from approximately 0.84 in ‘Dolanka’, DC109, DC182, DC233, DC295, DC359, DC704, DC717, and DC722; 0.82 in DC97, DC265, DC432, DC701, and DC724; 0.8 in DC522 and DC726; to 0.745 in DC728 (Figure 4A). Drought stress altered these values, which ranged from 0.84 down to 0.692. Eight genotypes (DC109, DC182, DC265, DC432, DC701, DC704, DC722, and DC724) exhibited significant reductions in Fv/Fm, with the largest declines observed in DC109, DC265, DC701, and DC722. Conversely, DC728 showed a 5% increase in Fv/Fm under drought.

The Performance Index (PI) for control plants spanned 0.67 to 3.03 and fell to 0.46–2.40 under drought (Figure 4B). Nearly all genotypes experienced significant PI declines, except DC233, DC704, DC717, and DC728; DC717 even increased by approximately 0.6 units. The strongest inhibitory responses (up to an 80% PI reduction) occurred in ‘Dolanka’, DC97, DC109, DC182, DC265, DC359, DC432, DC701, DC720, and DC722, with DC265 showing the greatest decrease.

**Figure 5:**
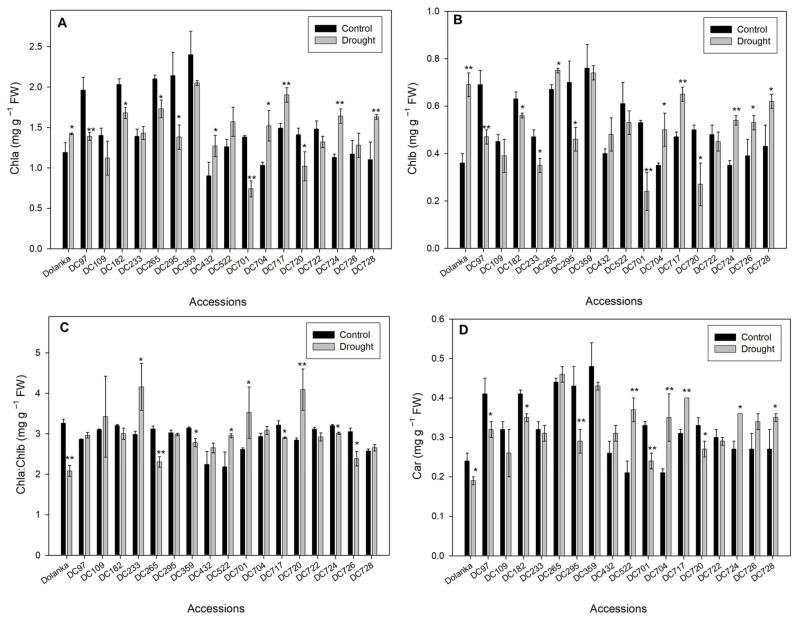
Content of chloroplastic pigments in the leaves of 18 carrot genotypes cultivated under control and stress conditions. (**A**) chlorophyll a (Chl a); (**B**) chlorophyll b (Chl b); (**C**) ratio of chlorophyll a and chlorophyll b (Chl a:Chl b), and (**D**) carotenoids (Car). Measurements were performed on 150-day-old plants. Statistically significant differences in stressed plants compared to controls are * *p* ≤ 0.1 and ** *p* ≤ 0.05.

Photosynthetic pigments analysis revealed substantial genotype-specific differences in chlorophyll a (Chl *a*), chlorophyll b (Chl *b*), their ratios, as well as carotenoid (Car) contents (Figure 5). In control leaves, Chl *a* ranged from 0.90 mg g^−1^ FW (DC432) to 2.40 mg g^−1^ FW (DC359). Under drought, Chl *a* and Chl *b* decreased markedly in DC97, DC295, and DC701, and slightly in DC182 and DC720. No change in these pigments occurred in DC109, DC233, DC522, DC722, or DC726. ‘Dolanka’ DC704, DC717, DC724, and DC728 accumulated more Chl *a* and Chl *b*. Differential response of leaves to stress reflected by various Chl *a* and Chl *b* accumulation was observed in DC432 and DC726. Drought stress also modified the Chl *a* and Chl *b* ratio in a genotype-specific manner. It declined in ‘Dolanka’, DC265, DC359, DC717, DC724, and DC726 but increased in DC233, DC522, DC701, and DC720. Car content rose under drought in DC522, DC704, DC717, DC724, and DC728, decreased in ‘Dolanka’, DC97, DC182, DC295, DC701, and DC720, and remained stable in the other accessions.

### 2.3. Impact of Drought on Seed Germination

Further analyses aimed to determine the effect of soil drought applied during the vegetative stage of development on the parameters related to the germination potential of formed seeds. The stressor significantly interacted with the genotypes to affect nearly all seed germination traits, except the CUG (Table 1). Germination capacity differed significantly between control and drought-treated seeds in ten genotypes. Three genotypes (DC182, DC233, and DC522) showed improved GC under drought, whereas seven (DC295, DC704, DC717, DC720, DC724, DC728, and ‘Dolanka’) performed better under well-watered conditions. Regarding AS, DC182 and DC728 produced fewer abnormal seedlings under drought compared to controls, while DC295, DC704, DC717, DC720, DC724, DC726, and ‘Dolanka’ exhibited higher frequencies of abnormal seedlings under stress. NGS (non-germinating seeds) responded variably: drought-stressed seeds of DC522 and DC726 had lower values than controls, but DC295, DC704, and DC720 showed the opposite pattern. Germination dynamics also revealed genotype-dependent responses to drought. MGT (mean germination time) was shorter under drought for DC97, DC522, and DC722, and longer for DC109, DC182, DC233, DC265, DC704, DC720, DC728, and ‘Dolanka’. Finally, the GI (germination index) improved under drought in DC97, DC522, and DC726, while it decreased in DC109, DC233, DC265, DC704, DC720, and ‘Dolanka’.

Comprehensive data on the effects of drought stress on the investigated traits, as well as the detailed responses of individual genotypes, are available in the Supplementary Materials (Tables S1–S6).

## 3. Discussion

Drought is among the most severe environmental stressors affecting reproductive success and limiting crop productivity, with serious implications for global food security [56,57,58]. Water deficit disrupts essential physiological processes [25], alters biochemical pathways—including enzymatic activities and oxidative-stress responses [35]—and triggers morphological adaptations such as reduced leaf area and enhanced root growth [59]. Although these adaptive mechanisms enable plant survival under transient stress, prolonged or intense drought ultimately reduces yield and quality and, in extreme cases, inhibits overall plant development [60]. The degree of drought tolerance depends primarily on stress intensity and duration, the plant’s developmental stage and physiological status, as well as species- or genotype-specific traits [61]. Notably, plants are most vulnerable during the critical stage of development–reproduction.

To mitigate the adverse effects of drought, plants accumulate osmoprotectants, which guard against dehydration and stabilize proteins and cellular structures. In carrot, osmotic adjustment has previously been shown to play a critical role in drought tolerance [55,62]. A prominent osmolyte in this context is the amino acid proline, which is widely used as a marker of osmotic stress [63]. The pronounced increase in proline content observed in most carrot genotypes following drought treatment (Figure 1) reflects activation of osmoprotective mechanisms. These findings confirm that proline is an effective early indicator of stress responses in carrot. Notably, DC522 and DC432 accessions exhibited more than a 10-fold rise in proline levels under drought, demonstrating strong adaptive capacity. In contrast, ‘Dolanka’ and DC359 failed to increase proline levels, indicating drought susceptibility and suggesting impaired osmoprotection mechanisms.

Drought stress induces excessive production of reactive oxygen species (ROS), disrupting the balance between ROS generation and the cellular mechanisms responsible for their neutralization [64]. Under normal conditions, ROS are maintained at low concentrations and play important roles in signal transduction and in regulating most processes related to plant growth and development [65]. However, in excess, singlet oxygen (^1^O_2_), superoxide anion radical (O_2_●^−^), hydrogen peroxide (H_2_O_2_), and hydroxyl radical (●OH) can damage cellular components, including membranes, pigments, proteins, nucleic acids, and lipids [66]. To counter oxidative stress, plants increase the activity of antioxidant enzymes, such as catalase (CAT), guaiacol peroxidase (GPOX), which detoxifies H_2_O_2_ using guaiacol as a substrate, and glutathione reductase (GR), which regenerates reduced glutathione (GSH) from its oxidized form (GSSG). As literature data show, antioxidant responses are related to tolerance/sensitivity to abiotic stresses in carrot [67].

Our results reveal genotype-specific changes in ROS metabolism under drought (Figure 2), suggesting diverse defense strategies among carrot genotypes. In DC726, DC724, DC722, and DC295, the simultaneous increase in both H_2_O_2_ and MDA (Figure 2A and Figure 3) indicates severe oxidative damage; the absence of a matching increase in CAT and GPOX activities (Figure 2B,C) suggests insufficient detoxification capacity and thus drought sensitivity. By contrast, DC522 and DC720 accessions showed a coordinated increase in H_2_O_2_ levels alongside CAT, GPOX, and GR activities (Figure 2), with stable or reduced MDA levels (Figure 3), denoting an efficient antioxidant network that mitigates lipid peroxidation. However, elevated enzyme activities do not uniformly equate to tolerance. DC233 and DC717 exhibited high antioxidant enzyme activities yet accumulated MDA (Figure 2 and Figure 3), implying that despite robust ROS-scavenging capacity, the metabolic costs or signaling intensity of stress exceed protective benefits. Similarly, DC432 and DC109 activated antioxidants but still show MDA accumulation, suggesting moderate tolerance. Interestingly, genotypes DC182 and DC728 displayed increased H_2_O_2_ without substantial MDA build-up or enzyme induction (Figure 2 and Figure 3), suggesting that H_2_O_2_ may function primarily as a signaling molecule triggering downstream adaptive responses rather than being directly detoxified. In DC97 and DC704, effective ROS management kept H_2_O_2_ and MDA at control levels under drought (Figure 2 and Figure 3), demonstrating prevention of lipid peroxidation with only moderate enzyme induction—an energy-conserving strategy. DC359 and DC265 accessions achieved tolerance through broad upregulation of all antioxidant enzymes, while in DC701, elevated CAT and GR activities alone sufficed for protection. The ‘Dolanka’ cultivar maintained stable ROS-related parameters, reflecting intrinsic drought tolerance.

A limitation of the presented study is that ROS scavenger levels and antioxidant enzyme activities were measured exclusively in leaf tissues, which primarily reflect systemic oxidative stress responses in vegetative organs. However, the redox status and ROS metabolism in reproductive structures–including flowers, umbels, and developing seeds–may differ substantially due to distinct metabolic demands, developmental programs, and tissue-specific antioxidant defense systems. Seeds synthesize their own autonomous antioxidant systems during embryogenesis and maturation, which do not necessarily mirror maternal leaf antioxidant profiles. Therefore, direct correlations between leaf antioxidant capacity and seed germination or seedling establishment should be interpreted with appropriate caution, recognizing that the observed relationships likely reflect integrated maternal effects transmitted through systemic signaling, resource allocation, and seed provisioning pathways rather than direct tissue-to-tissue transfer of antioxidant capacity. Future research should prioritize biochemical and molecular analyses of reproductive tissues to determine whether the antioxidant defense patterns observed in leaves correspond to those regulating seed development, maturation, and germination capacity under drought stress. Such tissue-specific analyses would provide more definitive mechanistic insight into how maternal redox homeostasis influences offspring vigor and stress tolerance in carrot.

Drought-induced declines in leaf water content suppress transpiration, raise leaf temperature, and promote wilting, impairing photosynthesis by damaging membranes, degrading chlorophyll pigments, and inhibiting PSII activity [66]. Our data show that DC728, DC717, and DC233 accessions maintained photosynthetic performance under drought, which can be supported by high pigment content (Figure 5) and PSII efficiency (Figure 4). In DC728 and DC717, increased carotenoid levels likely stabilized thylakoid membranes, delaying senescence rather than solely reflecting tolerance [68].

Another scenario is possible for the DC728 genotype; under drought, proline accumulation (Figure 1) further enhances antioxidant enzyme activity, scavenges excess H_2_O_2_ (Figure 2), and safeguards thylakoid membranes, as indicated by unchanged MDA content (Figure 3) and improved PSII activity (Figure 4) [69,70]. Conversely, DC97, DC726, DC265, DC720, DC701, DC295, and DC182 genotypes suffered pigment loss (chlorophyll and carotenoids) and reduced PSII efficiency, indicating photosynthetic sensitivity. In DC724, DC722, DC522, DC432, and DC109, pigment retention did not prevent photosynthetic impairment, likely due to ROS-induced disruption of electron transport despite stable chlorophyll content (Figure 2 and Figure 3). Meanwhile, in ‘Dolanka’, DC704, and DC359, photosynthesis declined without significant ROS accumulation, suggesting that factors beyond oxidative stress—such as stomatal closure or metabolic shifts—limit carbon fixation under drought.

Drought restricts the water required for seed germination and embryo development, resulting in deformities and increased abnormal seedling rates. Therefore, germination traits serve as key indicators of drought stress impacts during the generative phase. Previous studies have confirmed the detrimental effects of drought on seed development and viability in carrot and other crops [55,71,72,73].

Germination capacity (GC), the incidence of abnormal seedlings (AS), and the proportion of non-germinated seeds (NGS) reflect drought-induced reductions in seed quality, and carrot genotypes show pronounced variation in response, pointing to a complex genotype–environment interaction (Table 1). ’Dolanka’, DC717, DC724, DC728, DC295, DC704, and DC720 accessions were highly sensitive to water deficit, exhibiting reduced GC, developmental abnormalities, and, in the latter three genotypes, increased proportion of non-germinated seeds NGS. These adverse effects likely result from impaired photosynthetic processes observed in ‘Dolanka’, DC724, DC295, DC704, and DC720 (Figure 4), which limit assimilate supply and its mobilization to developing seeds [28].

Conversely, seeds of DC182 and DC233 germinated even more effectively under drought stress, which may indicate an adaptive response involving the production of seeds with enhanced viability. However, both showed reduced germination dynamics, potentially as a defense mechanism, delaying germination under unfavorable conditions. Similar strategies may be present in DC109 and DC265, which retained final germination capacity and morphology despite reduced germination speed. In DC726, drought had minimal impact on most germination traits, but the presence of developmental anomalies excludes it from being classified as drought tolerant. Genotypes DC97 and DC522 showed shortened mean germination time (MGT) and increased germination index (GI), suggesting rapid and efficient germination under drought. Notably, DC522 was the only genotype to exhibit improved values in all germination speed-related parameters under stress, indicating high drought tolerance. Furthermore, the unchanged coefficient of uniformity of germination (CUG) across all genotypes indicates synchronous germination even under adverse conditions.

## 4. Materials and Methods

### 4.1. Plant Material and Experimental Design

Eighteen carrot accessions (*Daucus carota* L.) were used in this study, including seventeen breeding male-sterile lines (DC97, DC109, DC182, DC233, DC265, DC295, DC359, DC432, DC522, DC701, DC704, DC717, DC720, DC722, DC724, DC726, and DC728) and one registered fertile cultivar, ‘Dolanka’. The plant material was provided by the Polish horticultural breeding and seed company PlantiCo—Hodowla i Nasiennictwo Ogrodnicze Zielonki Sp. z o.o. (Raciborowice, Poland). The breeding lines were developed through multiple breeding crosses and are not derived from the traditional cultivar ‘Dolanka’, which was included in this study solely as a reference due to its wide use and historical importance as a Polish open-pollinated population. The experimental lines exhibit considerable genetic diversity, making them suitable for evaluating genotype-specific responses to drought during the reproductive stage. The male-sterile breeding lines are part of ongoing breeding programs aimed at developing new carrot hybrids with improved agronomic and quality traits. They are selected not only for their yield potential, root morphology, and nutritional composition but also for their enhanced tolerance to biotic and abiotic stresses. Their use supports the development of cultivars better adapted to current and future environmental challenges.

Carrot stecklings were transplanted into 12-L plastic containers filled with 10 kg of a substrate consisting of garden soil, sand, and peat mixed in a 1:1:1 ratio. The substrate had a nutrient content of N:P:K at approximately 1:2:2 and pH 6.5–7.0. Four stecklings were planted per container. The experiment followed a randomized block design with three replicates per treatment. For each of the 18 accessions, six containers (24 stecklings) were used—three under drought stress and three as controls. The experiment was conducted in a greenhouse at the University of Agriculture in Krakow, Poland, under controlled conditions. During the early growth phase, the greenhouse was maintained at approximately 13 °C at night and 17 °C during the day. Supplemental lighting extended the photoperiod to 16 h of light. Once the seed stalks emerged, the temperature increased to 21 °C at night and 27 °C during the day. Standard carrot cultivation practices, including fertilization, pest and disease control, and routine care, were applied throughout the experiment.

Before the drought stress application, all plants were irrigated equally with the amount of water required to maintain optimal soil moisture at approximately 25% volumetric moisture content (VWC). This value represents the volume of water per volume of soil and was measured using calibrated soil moisture sensors (see below). Drought stress was initiated once flower stalks had developed, but before the onset of flowering. A drip irrigation system was used to maintain optimal soil moisture conditions in the control group, while drought-stressed plants received only one-third of the water volume administered to controls.

Soil moisture was monitored using a Theta Probe (ML3) and a soil moisture sensor (SM150 with HH2 handheld reader), both from Delta-T Devices (Cambridge, UK). Measurements were taken at a depth of 6 cm at four different points in each container. The probes were calibrated specifically for mineral soils to ensure accurate volumetric moisture content (VWC) readings. In drought-stressed containers, soil moisture was maintained at approximately 8–9% (*v*/*v*). Seven weeks after the initiation of the drought treatment, physiological and biochemical measurements were conducted on carrot leaves. Chlorophyll *a* fluorescence was measured on the first fully expanded, mature leaves from the middle section of the shoot. After the measurements, the same leaves were immediately frozen in liquid nitrogen and stored at −80 °C for further biochemical analyses.

Seed-bearing umbels were harvested manually at full seed maturity. Each replicate was collected separately in labeled paper bags and air-dried at room temperature for six weeks. After drying, the seeds were manually threshed and cleaned using a set of sieves.

### 4.2. Assessment of Biochemical Parameters

#### 4.2.1. Quantification of Proline by LC-MS/MS

The concentration of proline was measured using a liquid chromatography–tandem mass spectrometry (LC-MS/MS) system, as previously described by Ledwożyw-Smoleń et al. [74], with minor modifications. The analytical platform consisted of an Ultimate 3000 HPLC system (Thermo Fisher Scientific, Waltham, MA, USA) coupled to a 4500 QTRAP mass spectrometer (Sciex, Framingham, MA, USA). Proline standards were procured from Sigma-Aldrich (Merck KGaA, Darmstadt, Germany). For sample preparation, approximately 50 mg of freeze-dried leaf tissue was weighed into 7 mL polypropylene tubes and treated with 5 mL of 75% ethanol. The mixture was vortexed and subjected to 10 min of ultrasonic extraction at 25 °C. Next samples were vortexed and centrifuged at 4500 rpm for 5 min, and the supernatant (1 mL) was filtered through a syringe filter (⌀ 25 mm, 0.2 µm pore size) into a 1.5 mL Eppendorf tube. After filtration, 1 mL of the extract was transferred to LC vials for analysis. The chromatographic conditions and LC-MS/MS parameters were as described in Ledwożyw-Smoleń et al. [74]. Data acquisition and analysis were performed using Analyst 1.7 software (HotFix 3) and MultiQuant 3.0.3 (version 3.0.31721.0).

#### 4.2.2. Determination of Antioxidant Enzyme Activity and H_2_O_2_ Measurements

To analyze the activity of selected antioxidant enzymes, fresh leaf samples (0.25 g) were extracted with 100 mM phosphate buffer (pH 7.5) containing 1 mM EDTA (ethylenediaminetetraacetic acid) and 1% PVP (polyvinylpyrrolidone). Samples were homogenized and centrifuged for 15 min (4500 rpm, 5 °C). The collected supernatant was further centrifuged (20 min, 10,000 rpm, 2 °C) and stored at −80 °C until analyses. In the obtained extracts, the activity of CAT, POX, and GR was determined. Catalase (hydrogen–peroxide/hydrogen–peroxide oxidoreductase; EC 1.11.1.6) activity was measured according to Beers and Sizer [75] with modifications. Reaction mixture of 3 mL contained 100 mM phosphate buffer (pH 7.5), 200 mM H_2_O_2,_ and enzyme extract. Absorbance of the mixture was measured at 240 nm as the change of H_2_O_2_ concentration (ε = 26.6 mM^−1^ cm^−1^) occurring 45 and 60 s after the addition of enzyme extract. Specific activity of CAT was expressed as μmol of oxidized H_2_O_2_ mg protein^−1^ min^−1^ (U mg^−1^ protein).

Peroxidase (guaiacol/hydrogen–peroxide oxidoreductase; EC 1.11.1.7) activity was analyzed according to Reuveni et al. [76] with further modifications. A total volume of 2 mL of reaction mixture contained 15 mM phosphate buffer (pH 6.5), 10 mM guaiacol, enzyme extract, and 1 mM H_2_O_2_ as an initiating factor. The increase in absorbance of oxidized guaiacol (ε = 26.6 mM^−1^ cm^−1^) was measured at 470 nm. Enzymatic activity of POX was expressed as μmol of oxidized guaiacol mg protein^−1^ min^−1^ (U mg^−1^ protein).

Glutathione reductase (EC 1.8.1.7) activity was determined spectrophotometrically by monitoring the decrease in absorbance at 340 nm, which corresponds to the oxidation of NADPH, as described by Carlberg and Mannervik [77] with slight modifications. The assay was conducted at 25 °C in a final volume of 3.0 mL, comprising 0.1 M phosphate buffer (pH 7.8) prepared from KH_2_PO_4_ and Na_2_HPO_4_ (2.1 mL), 0.5 mM oxidized glutathione (GSSG; 300 µL), 0.2 mM NADPH (300 µL), and 300 µL of crude enzyme extract. Both GSSG and NADPH solutions were freshly prepared in phosphate buffer before use. The reaction was initiated by the addition of NADPH, and the absorbance change at 340 nm was recorded for 2–3 min using an extinction coefficient of 6.2 mM^−1^ cm^−1^. Enzyme activity was calculated and expressed as nanomoles of NADPH oxidized per minute per milligram of protein (U mg^−1^ protein).

The concentration of H_2_O_2_ was analyzed, as described previously [78]. The results are presented as µmol H_2_O_2_ per gram of fresh weight.

#### 4.2.3. Determination of Malondialdehyde (MDA) Content

Lipid peroxidation was assessed by quantifying MDA, a key marker of oxidative membrane damage. The protocol was based on the thiobarbituric acid (TBA) assay originally developed by Hodges et al. [79], with slight procedural adjustments as described previously by Kućko et al. [80]. Leaf samples were collected and immediately subjected to lyophilization. The lyophilized material was then homogenized using a tissue grinder to obtain a fine powder, and approximately 200 mg of powder was homogenized in 2 mL of 0.1% (*w*/*v*) trichloroacetic acid (TCA). The homogenate was centrifuged at 10,000 rpm for 10 min at 4 °C. Subsequently, 1 mL of the supernatant was combined with an equal volume of 0.5% (*w*/*v*) TBA in 20% (*w*/*v*) TCA. The reaction mixture was incubated at 95 °C for 30 min and immediately cooled on ice to stop the reaction. After centrifugation (10,000 rpm, 10 min), the absorbance of the supernatant was recorded at 532 nm and corrected for nonspecific turbidity by subtracting the absorbance at 600 nm.

### 4.3. Assessment of Physiological Traits

Chlorophyll *a* fluorescence was determined using a Handy-PEA (Hansatech Ltd., King’s Lynn, UK) fluorimeter. Measurements were taken on 10 fully developed leaves for each genotype and treatment. After 30 min of dark adaptation by the leaf-clips, the leaf tissue was exposed to red light (650 nm) flash (duration: 1 s, intensity: 3500 µmol m^−2^ s^−1^) during which the fluorescence signal was recorded. The obtained data involves the maximum quantum yield of photosystem II (Fv/Fm) and Performance Index (PI) parameters.

The content of photosynthetic pigments—Chl *a*, Chl *b,* and Car was measured (in triplicate) with the use of the method described by Lichtenthaler [81]. Cold extraction of pigments from leaves using 80% acetone (Chempur, Piekary Śląskie, Poland) as a solvent was performed. Then, samples were centrifuged (4800 rpm at 4 °C for 15 min; Rotina 380R, Hettich, Germany), and the supernatant was transferred to clean plastic test tubes. After that, the pellet was again ground with acetone, and the samples were centrifuged again. Supernatants from both extractions were combined, and the absorbance at 470, 646, and 663 nm was measured with a UV–Vis spectrophotometer (U–2900, Hitachi, Tokyo, Japan). The content of Chl *a*, Chl *b,* their ratio, as well as Car, was calculated according to equations described by Porra [82] and expressed in mg of pigment per 100 g of FW.

### 4.4. Assessment of Seed Germination

In the test, germination capacity, as the percentage of normal seedlings (GC), the percentage of abnormal seedlings (AS), and the percentage of non-germinated seeds (NGS) were assessed on day 14, according to the “International Rules for Seed Testing” [83]. Seedling quality was determined following the guidelines in the “Seedling Evaluation Handbook” [84]. Furthermore, we calculated parameters reflecting germination speed and uniformity: mean germination time (MGT, days), coefficient of velocity of germination (CVG, %), germination index (GI, seeds day^−1^), and coefficient of uniformity of germination (CUG, day^−2^). Germinated seeds—defined as those having a radicle of at least 2 mm—were counted daily at the same time for 14 consecutive days. The following formulas were applied to calculate the respective parameters [1,2,3,4]:

(1) $$MGT=\frac{\sum \left(D\times N\right)}{\sum N},$$

where D is the number of days since the start of the test, and N is the number of seeds germinated on day D [85],

(2) $$CVG=\frac{\sum N}{\sum \left(D\times N\right)}\times 100,$$

where D and N as defined above [86],

(3) $$MGT=\left(14\times N_{1}\right)+\left(13\times N_{2}\right)+\cdots +\left(1\times N_{14}\right),$$

where N_1_, N_2_, …, N_14_ are the numbers of seeds germinated on each respective day, and the values 14, 13, …, 1 are assigned weights corresponding to the number of germinated seeds on each respective day [87],

(4) $$CUG=\frac{\sum N}{\sum (MGT-{D)}^{2}\times N\;}\;$$

where D and N are as defined above [88,89].

### 4.5. Statistical Analysis

The biochemical data are results of the analysis performed in three biological replicates; each biological sample was examined two times. The data represent the mean ± standard error (SE). All data were analyzed using two-way analysis of variance (ANOVA) with genotype and treatment (control vs. drought) as fixed effects. The model included the interaction term genotype × treatment to test for differential drought responses among genotypes. When significant effects were detected (*p* < 0.05), mean separation was performed using Fisher’s Least Significant Difference (LSD) post hoc test. Significant differences among means are indicated by different letters in tables and figures, and asterisks indicate significant differences between control and drought-stressed plants. All statistical analyses were conducted using Statistica 13.3 software (TIBCO Software Inc., Palo Alto, CA, USA).

## 5. Conclusions

**Figure 6:**
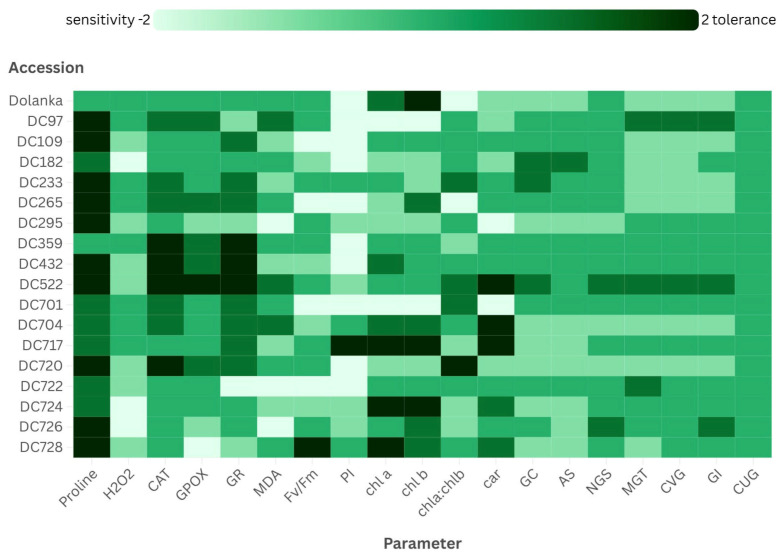
Summary of drought tolerance in carrot genotypes. Heatmap visualization of drought tolerance parameters across carrot accessions. Median values for each measured parameter are displayed using a color gradient from green (high drought tolerance) to white (low drought tolerance), facilitating the identification and selection of drought-tolerant cultivars. For further details, see the Discussion Section.

This study demonstrates that drought tolerance in carrot is a multifactorial trait, involving oxidative stress responses, photosynthetic efficiency, and seed germination capacity. The substantial genotypic variation observed allowed us to distinguish both drought-tolerant and drought-sensitive accessions, as summarized in Figure 6.

Among the analyzed genotypes, DC522 exhibited the highest level of drought tolerance, characterized by enhanced antioxidant enzyme activities, increased accumulation of chloroplast pigments, and even improved germination performance under drought compared to control conditions. The second most tolerant genotype, DC717, showed superior photosynthetic performance and chlorophyll content; however, the seeds produced by this genotype were highly sensitive to drought and are therefore not recommended as sowing material. Desirable seed quality under drought stress was also observed in DC359, DC432, and DC701, which additionally exhibited efficient enzymatic antioxidant systems during the generative growth stage. In contrast, DC295, due to its weak antioxidant defense, reduced photosynthetic capacity, and impaired germination and seedling development under drought, is not suitable for cultivation in environments prone to water deficits. The findings presented in this study provide valuable insights for future carrot breeding programs aimed at enhancing drought tolerance.

The identification of drought-tolerant genotypes, particularly DC522, offers valuable material for carrot breeding programs targeting water-limited environments. Key physiological and biochemical traits—such as enhanced antioxidant efficiency, pigment stability, and superior germination performance—can serve as preliminary selection criteria in future screening efforts. Further multi-environment trials and genetic analyses are required to validate the heritability and stability of these traits. Such studies will facilitate the integration of these indicators into marker-assisted selection strategies for developing drought-tolerant carrot cultivars.

## Figures and Tables

**Table 1:** The influence of drought stress on the germination traits of 18 carrot accessions.

Accession	Growing Condition	GC (%)	AS (%)	NGS (%)	MGT (Day)	CVG (%)	GI (Seed Day^−1^)	CUG (Day^−2^)
‘Dolanka’	control	97.0 a–d	1.0 jk	2.0 d–h	3.66 o	27.46 c	310.8 cde	1.78 ns
	drought	72.5 m *	25.5 a *	2.0 d–h	4.13 g–j *	24.27 g–k *	287.8 h–o *	1.25 ns
DC97	control	95.5 a–f	0.0 k	4.5 b–e	4.58 b	21.86 p	259.0 stu	0.72 ns
	drought	94.5 c–g	2.5 h–k	3.0 b–h	4.02 i–m *	24.89 d–i *	290.0 h–o *	1.22 ns
DC109	control	96.0 a–e	0.5 jk	3.5 b–g	3.92 klm	25.50 def	293.3 g–l	1.35 ns
	drought	93.0 d–h	3.0 g–k	4.0 b–f	5.63 a *	17.76 q *	209.5 w *	0.56 ns
DC182	control	88.5 ijk	9.0 bcd	2.5 c–h	4.04 i–l	24.78 e–j	290.5 h–n	0.66 ns
	drought	96.5 a–d *	2.5 h–k *	1.0 fgh	4.37 cde *	22.86 m–p *	278.5 m–q	1.01 ns
DC233	control	92.0 e–i	3.5 g-j	4.5 b–e	3.47 pr	28.82 b *	313.3 bcd	1.23 ns
	drought	97.0 a–d *	1.5 ijk	1.5 e–h	3.94 klm *	25.40 def *	298.5 e–i *	1.24 ns
DC265	control	99.5 a	0.0 k	0.5 gh	3.28 r *	30.48 a	334.3 a	1.27 ns
	drought	97.5 ab	2.5 h–k	0.0 g	4.16 ghi *	24.04 h–l *	290.5 h–n *	1.59 ns
DC295	control	97.5 abc	1.5 ijk	1.0 fgh	4.22 e–h	23.71 j–m	286.0 i–p	1.41 ns
	drought	88.0 ijk *	6.0 d–g *	6.0 b *	4.17 ghi	24.01 h–l	274.3 pqr	2.22 ns
DC359	control	97.5 abc	1.5 ijk	1.0 fgh	3.86 mn	25.92 d	304.0 d–g	3.15 ns
	drought	97.5 abc	2.0 ijk	0.5 gh	4.02 i–m	24.95 d–i	297.8 e–j	2.33 ns
DC432	control	94.4 c–g	2.5 h–k	3.0 b-h	4.57 b	21.89 p	264.8 rst	1.37 ns
	drought	91.0 ghi	3.0 g–k	6.0 b	4.53 bc	22.11 op	257.0 tu	2.00 ns
DC522	control	85.5 jkl	4.5 f–i	10.0 a	4.47 bcd	22.38 nop	249.0 u	1.77 ns
	drought	96.5 a–d *	2.5 h–k	1.0 fgh *	3.94 klm *	25.36 d–g *	299.8 e–h *	1.32 ns
DC701	control	96.5 a–d	0.5 jk	3.0 b-h	3.90 lm	25.66 de	296.0 f–k	1.86 ns
	drought	94.0 c–g	1.5 ijk	4.5 b-e	3.90 lm	25.64 de	291.3 g–m	1.62 ns
DC704	control	98.0 abc	0.0 k	2.0 d-h	3.45 qr	29.02 b	321.0 abc	2.33 ns
	drought	89.0 hij *	5.5 e–h *	5.5 bc *	3.70 no *	27.03 c *	299.3 e–i *	1.37 ns
DC717	control	95.0 b–g	4.5 f–i	0.5 gh	4.09 h–k	24.49 f–k	294.3 g–l	3.55 ns
	drought	84.5 kl *	12.0 b *	3.5 b-g	4.17 f–i	24.00 i–l	284.3 k–q	3.01 ns
DC720	control	99.0 ab	0.0 k	1.0 fgh	3.44 qr	29.08 b	324.8 ab	3.66 ns
	drought	83.5 l *	11.0 bc *	5.5 bc *	4.13 g–j *	24.20 h–l *	277.3 n–r *	1.93 ns
DC722	control	91.5 f–i	6.0 d–g	2.5 c–h	4.47 bcd *	27.51 c	310.3 cde	1.80 ns
	drought	93.0 d–h	3.0 g–k	4.0 b–f	3.56 opr *	28.12 bc	309.3 c–f	1.52 ns
DC724	control	91.5 f–i	7.0 d–f *	1.5 e–h	4.27 efg	23.45 k–n	282.3 l–q	1.92 ns
	drought	86.0 jkl *	12.0 b *	2.0 d–h	4.35 de	23.02 l–o	277.0 o–r	2.25 ns
DC726	control	91.5 f–i	3.5 g–j	5.0 bcd	4.02 i–m	24.89 d–i	284.0 k–q	3.44 ns
	drought	89.00 hij	10.5 bc *	0.5 gh *	3.98 j–m	25.10 d–h	299.3 e–i *	2.42 ns
DC728	control	97.0 a–d	0.5 jk	2.5 c–h	4.16 ghi	24.05 h–l	284.8 j–q	2.00 ns
	drought	88.0 ijk *	8.0 cde *	4.0 b–f	4.34 def *	23.02 l–o	271.5 qrs	1.97 ns

GC—germination capacity; AS—abnormal seedlings; NGS—non-germinating seeds; MGT—mean germination time; CVG—coefficient of velocity of germination; GI—germination index; CUG—coefficient of uniformity of germination. Values marked with the same letters do not differ significantly at the assumed significance level of *p* < 0.05, according to Fisher’s LSD test; ns—not significant. Statistically significant difference in stressed plants compared to controls is * *p* ≤ 0.05.

## Data Availability

Data are contained within the article.

## Supplementary Materials

The following supporting information can be downloaded at: https://www.mdpi.com/article/10.3390/ijms262110642/s1.
