# Supplementary material for: Genotype-Specific Responses to Drought During Seed Production in Carrot: Biochemical, Physiological, and Seed Quality Evaluation
Source: Int J Mol Sci. 2025 Oct 31;26(21):10642. doi: 10.3390/ijms262110642 (PMC12608256; doi:10.3390/ijms262110642)
Supplement: Supplementary file 1 [file ijms-26-10642-s001.zip › ijms-3908685-supplementary.pdf]

Supplementary Materials

Table S1. The effect of drought stress on biochemical parameters.

Growing Conditions	Proline (mg kg⁻¹ DW)	H_2O_2 (µmol mg⁻¹ FW)	CAT (U mg⁻¹ protein)	GPOX (U mg⁻¹ protein)	GR (U mg⁻¹ protein)	MDA (nmol mL⁻¹)
**Control**	41.3 b	0.15 b	0.04 b	0.032 b	0.17 b	2.8 b
**Drought**	231.0 a	0.25 a	0.10 a	0.039 a	0.19 a	3.9 a

MDA—malondialdehyde; CAT—catalase; GPOX—glutathione peroxidase; GR—glutathione reductase. Values marked with the same letters do not differ significantly at the assumed significance level of $p < 0.05$, according to Fisher's LSD test; ns—not significant.

Table S2. Biochemical parameters of different carrot genotypes.

Genotype	Proline (mg kg⁻¹ DW)	H_2O_2 (µmol mg⁻¹ FW)	CAT (U mg⁻¹ protein)	GPOX (U mg⁻¹ protein)	GR (U mg⁻¹ protein)	MDA (nmol mL⁻¹)
Dolanka	161.1 cd	0.20 a–d	0.05 h	0.021 e–g	0.14 hi	2.0 f
DC97	165.6 b–d	0.17 cd	0.07 e–h	0.050 bc	0.21 d–f	3.4 de
DC109	190.4 ab	0.22 a–c	0.07 e–h	0.046 c	0.25 b–d	4.4 bc
DC182	99.5 gh	0.21 a–c	0.06 gh	0.050 bc	0.19 fg	3.1 e
DC233	174.1 bc	0.21 a–c	0.10 a–d	0.031 d	0.20 ef	3.7 d
DC265	186.9 a–c	0.19 b–d	0.09 b–e	0.057 b	0.20 ef	3.1 e
DC295	146.2 de	0.24 ab	0.05 h	0.029 de	0.20 ef	3.4 de
DC359	50.7 i	0.14 d	0.12 a	0.029 de	0.13 hi	1.9 f
DC432	190.6 ab	0.19 b–d	0.11 ab	0.018 f–h	0.09 jk	2.3 f
DC522	201.2 a	0.15 d	0.08 c–g	0.052 bc	0.15 g–i	4.9 ab
DC701	116.1 fg	0.17 cd	0.06 f–h	0.027 d–f	0.29 b	3.4 de
DC704	106.4 gh	0.17 cd	0.11 a–c	0.016 g–i	0.03 l	5.3 a
DC717	85.1 h	0.19 b–d	0.07 d–h	0.011 hi	0.06 kl	4.7 ab
DC720	133.3 ef	0,20 a–d	0.09 c–f	0.09 i	0.12 ij	1.9 g
DC722	87.6 h	0.22 a–c	0.05 h	0.046 c	0.35 a	3.5 de
DC724	95.7 gh	0.25 a	0.06 f–h	0.058 b	0.27 bc	2.4 f
DC726	140.3 d–f	0.21 a–c	0.05 h	0.022 e–g	0.17 f–h	4.0 cd
DC728	120.0 fg	0.21 a–c	0.05 h	0.072 a	0.24 de	3.4 de

MDA—malondialdehyde; CAT—catalase; GPOX—glutathione peroxidase; GR—glutathione reductase. Values marked with the same letters do not differ significantly at the assumed significance level of $p < 0.05$, according to Fisher's LSD test; ns—not significant.

Table S3. The effect of drought stress on photosynthetic apparatus parameters of carrots' leaves.

Growing Conditions	Fv/Fm	PI	Chl a (mg g^{-1} FW)	Chl b (mg g^{-1} FW)	Chl a:Chl b (mg g^{-1} FW)	Car (mg g^{-1} FW)
**Drought**	0.815 a	1.56 ns	1.43 ns	0.51 ns	3.01 ns	0.32 ns
**Control**	0.823 b	1.62 ns	1.50 ns	0.51 ns	2.92 ns	0.32 ns

Fv/Fm—maximum quantum efficiency of photosystem II photochemistry; PI—Performance Index; Chl *a*—chlorophyll a; Chl *b*—chlorophyll b; Chl *a*:Chl *b*—ratio of chlorophyll a and chlorophyll b; Car—carotenoids. Values marked with the same letters do not differ significantly at the assumed significance level of $p < 0.05$, according to Fisher's LSD test; ns—not significant.

Table S4. Photosynthetic apparatus parameters of carrots' leaves of different carrot genotypes.

Genotype	Fv/Fm	PI	Chl a (mg g^{-1} FW)	Chl b (mg g^{-1} FW)	Chl a:Chl b (mg g^{-1} FW)	Car (mg g^{-1} FW)
Dolanka	0.817 b–d	1.35 a–d	1.15 c–e	0.43 e–h	2.80 ab	0.22 ab
DC97	0.831 cd	1.78 d–g	1.24 ef	0.41 d–g	3.22 bc	0.27 d–f
DC109	0.820 b–d	1.43 c–e	1.03 bc	0.33 a–c	3.67 de	0.24 a–d
DC182	0.803 ab	0.95 ab	1.25 ef	0.39 d–g	3.81 e	0.28 ef
DC233	0.826 cd	1.81 d–g	1.14 c–e	0.32 ab	4.00 e	0.27 d–f
DC265	0.829 cd	1.66 d–g	1.42 gh	0.47 hi	3.56 c–e	0.34 h
DC295	0.831 cd	1.98 f–h	1.31 fg	0.38 c–f	4.95 f	0.29 fg
DC359	0.816 b–d	1.41 b–e	1.51 h	0.50 i	3.12 a–c	0.32 gh
DC432	0.808 a–c	1.40 a–e	0.86 a	0.33 a–c	2.67 a	0.21 a
DC522	0.823 b–d	1.65 d–f	1.15 c–e	0.44 g–i	2.67 a	0.24 a–d
DC701	0.818 b-d	1.49 c–e	0.90 ab	0.32 a	3.00 ab	0.23 a–c
DC704	0.838 d	2.35 h	1.22 d–f	0.42 d–h	2.89 ab	0.28 ef
DC717	0.829 cd	1.88 e–g	1.33 fg	0.44 f–h	3.06 ab	0.28 ef
DC720	0.816 b–d	1.72 d–g	1.15 c–e	0.37 b–d	3.23 b–d	0.27 d–f
DC722	0.829 cd	1.65 d–f	1.09 cd	0.37 b–d	2.89 ab	0.22 ab
DC724	0.813 bc	1.09 a–c	1.12 c–e	0.37 b–d	3.03 ab	0.25 b–e
DC726	0.819 b–d	2.14 gh	1.04 bc	0.38 c–e	2.84 ab	0.25 b–e
DC728	0.785 a	0.91 a	1.09 cd	0.41 d–g	2.71 a	0.25 b–e

Fv/Fm—maximum quantum efficiency of photosystem II photochemistry; PI—Performance Index; Chl *a*—chlorophyll a; Chl *b*—chlorophyll b; Chl *a*:Chl *b*—ratio of chlorophyll a and chlorophyll b; Car—carotenoids. Values marked with the same letters do not differ significantly at the assumed significance level of $p < 0.05$, according to Fisher's LSD test; ns—not significant.

Table S5. The effect of drought stress on carrot seeds germination traits.

Growing Conditions	GC (%)	AS (%)	NGS (%)	MGT (day)	CVG (%)	GI (seed day^{-1})	CUG (day^{-2})
**Control**	94.6 a	2.6 b	2.8 ns	3.95 b	25.61 a	294.6 a	1.98 a
**Drought**	90.6 b	6.4 a	3.0 ns	4.17 a	25.21 b	282.9 b	1.70 b

GC—germination capacity; AS—abnormal seedlings; NGS—non–germinating seeds; MGT—mean germination time; CVG—coefficient of velocity of germination; GI—germination index; CUG—coefficient of uniformity of germination. Values marked with the same letters do not differ significantly at the assumed significance level of $p < 0.05$, according to Fisher's LSD test; ns—not significant.

Table S6. Seed germination traits of different carrot genotypes.

Genotype	GC (%)	AS (%)	NGS (%)	MGT (day)	CVG (%)	GI (seed day^{-1})	CUG (day^{-2})
Dolanka	84.8 g	13.3 a	2.0 cde	3.89 ef	25.87 de	299.3 cd	1.52 c–f
DC97	95.0 bc	1.3 hi	3.8 abc	4.30 c	23.38 i	274.5 h	0.97 ef
DC109	94.5 bc	1.8 ghi	3.8 abc	4.78 a	21.63 j	251.4 j	0.96 ef
DC182	92.5 cde	5.8 de	1.8 cde	4.21 cd	23.82 hi	284.5 efg	0.83 f
DC233	94.5 bc	2.5 f–i	3.0 bcd	3.71 gh	27.11 bc	305.9 abc	1.23 def
DC265	98.5 a	1.3 hi	0.3 e	3.72 g	27.26 abc	312. 4 a	1.43 c–f
DC295	92.8 cde	3.8 efg	3.5 abc	4.19 cd	23.86 hi	280.1 fgh	1.81 cd
DC359	97.5 ab	1.8 ghi	0.8 de	3.94 e	25.43 ef	300.9 bcd	2.74 ab
DC432	92.8 cde	2.8 f–i	4.5 ab	4.55 b	22.00 j	260.9 i	1.69 cde
DC522	91.0 def	3.5 e–h	5.5 a	4.21 cd	23.87 hi	274.4 h	1.55 c–f
DC701	95.3 bc	1.0 i	3.8 abc	3.90 ef	25.65 ef	293.6 de	1.74 cde
DC704	93.5 cd	2.8 f–i	3.8 abc	3.57 i	28.03 a	310.1 ab	1.85 cd
DC717	89.8 ef	8.3 bc	2.0 cde	4.13 d	24.24 gh	289.3 ef	3.28 a
DC720	91.3 def	5.5 de	3.3 abc	3.79 fg	26.64 cd	301.0 bcd	2.79 ab
DC722	92.3 cde	4.5 ef	3.3 abc	3.60 hi	27.81 ab	309.8 ab	1.66 c–f
DC724	88.8 f	9.5 b	1.8 cde	4.31 c	23.23 i	279.6 gh	2.08 bc
DC726	90.3 ef	7.0 cd	2.8 bcd	4.00 e	25.00 fg	291.6 de	2.93 a
DC728	92.5 cde	4.3 ef	3.3 abc	4.25 c	23.54 hi	278.1 gh	1.99 bcd

GC—germination capacity; AS—abnormal seedlings; NGS—non–germinating seeds; MGT—mean germination time; CVG—coefficient of velocity of germination; GI—germination index; CUG—coefficient of uniformity of germination. Values marked with the same letters do not differ significantly at the assumed significance level of $p < 0.05$, according to Fisher's LSD test; ns—not significant.
